# The benefit of co-targeting PARP-1 and c-Met on the efficacy of radiotherapy in wild type BRAF melanoma

**DOI:** 10.3389/fmed.2023.1149918

**Published:** 2023-05-04

**Authors:** Malak Sabbah, Ahmad Najem, Christophe Vanderkerkhove, Fabien Kert, Younes Jourani, Fabrice Journe, Ahmad Awada, Dirk Van Gestel, Ghanem E. Ghanem, Mohammad Krayem

**Affiliations:** ^1^Laboratory of Clinical and Experimental Oncology (LOCE), Institut Jules Bordet, Université Libre de Bruxelles (ULB), Hôpital Universitaire de Bruxelles (H.U.B), Bruxelles, Belgium; ^2^Medical Physics Department, Institut Jules Bordet, Université Libre de Bruxelles (ULB), Hôpital Universitaire de Bruxelles (H.U.B), Brussels, Belgium; ^3^Oncology Medicine Department, Jules Bordet Institute, Université Libre de Bruxelles (ULB), Hôpital Universitaire de Bruxelles (H.U.B), Brussels, Belgium; ^4^Radiation Oncology Department, Institut Jules Bordet, Université Libre de Bruxelles (ULB), Hôpital Universitaire de Bruxelles (H.U.B), Brussels, Belgium

**Keywords:** melanoma, radiotherapy, WTBRAF, RTK inhibition, PARP inhibition

## Abstract

Melanoma is known to be a radioresistant cancer. Melanoma radioresistance can be due to several factors such as pigmentation, antioxidant defenses and high Deoxyribonucleic acid (DNA) repair efficacy. However, irradiation induces intracellular translocation of RTKs, including cMet, which regulates response to DNA damage activating proteins and promotes DNA repair. Accordingly, we hypothesized that co-targeting DNA repair (PARP-1) and relevant activated RTKs, c-Met in particular, may radiosensitize wild-type B-Raf Proto-Oncogene, Serine/Threonine Kinase (WTBRAF) melanomas where RTKs are often upregulated. Firstly, we found that PARP-1 is highly expressed in melanoma cell lines. PARP-1 inhibition by Olaparib or its KO mediates melanoma cell sensitivity to radiotherapy (RT). Similarly, specific inhibition of c-Met by Crizotinib or its KO radiosensitizes the melanoma cell lines. Mechanistically, we show that RT causes c-Met nuclear translocation to interact with PARP-1 promoting its activity. This can be reversed by c-Met inhibition. Accordingly, RT associated with the inhibition of both c-Met and PARP-1 resulted in a synergistic effect not only on tumor growth inhibition but also on tumor regrowth control in all animals following the stop of the treatment. We thus show that combining PARP and c-Met inhibition with RT appears a promising therapeutic approach in WTBRAF melanoma.

## 1. Introduction

Metastatic melanoma is a mutinous disease, often requiring drug combinations. Currently, a selective mutant BRAF/MEK inhibitor combination with immunotherapy shows a very good response (about 75%) and is largely used in the treatment of BRAF mutant melanoma patients (1). Non-BRAF mutant melanoma patients have limited treatment options with only 30% responding to tyrosine kinase (TK) inhibitors against growth factor receptors (2). In 2017, Hayward et al. published a Whole Genome Sequencing (WGS) comparing cutaneous, acral and mucosal subtypes that showed aberrations in receptor tyrosine kinase (RTK) pathways in about 49% of cutaneous melanoma and 42% of acral and mucosal melanomas, which is considered relatively high (3). As RTKs were found associated with resistance to MAPK inhibition, phenotype switch, invasion, metastases and relapses (4–7), targeting RTKs was considered of particular importance in melanoma, especially in the non-BRAF mutant melanoma subgroup. However, specific inhibition of RTKs demonstrated short/limited efficacy in the relevant melanoma subgroup despite all pre-clinical expectations. Nowadays, planned, published and ongoing trials include combinations of RTK inhibition with other treatment modalities underlining the importance of finding efficient treatments for non-BRAF mutant melanoma patients (8).

Melanoma is considered as a radioresistant tumor showing a broad shoulder in survival fraction assay. This is due to high melanoma cell proliferation capacities, efficient antioxidant defenses (e.g., glutathione and melanin pigment), poor cell differentiation, apoptosis abnormalities due to p53 attenuation, and efficient DNA repair machinery (9–11). However, radiotherapy as an adjuvant treatment for patients suffering from advanced disease reduces the risk of local and metastatic tumor recurrence (12). Furthermore, stereotactic radiosurgery (SRS) achieved tumor control (13, 14) and improved overall survival (OS) (13, 15, 16). For this reason, SRS alone was recommended for melanoma patients with limited brain metastases (up to 10) and tumor diameters that do not exceed 4 cm (17). Stereotactic body radiation therapy (SBRT) refers to high-dose fractionated radiotherapy (RT) that is particularly useful for patients with oligometastatic disease mainly involving lung, bone, liver, or adrenals. Also, radiotherapy is often used in palliative settings primarily for patients with pain, mass effect, tumor-related hemorrhage, and local irritation of skin or subcutaneous lesions (17).

Nowadays, cancer radiosensitizers that include novel targeted therapies, are considered as promising compounds to enhance oxidative stress and DNA damage (18). Accordingly, combining ionizing radiations (IR) with such radiosensitizers can be an opportunity to increase radiotherapy efficacy while minimizing toxicity to healthy surrounding tissues (19).

Particularly, IR-induced DNA single-strand breaks (SSBs) recruit poly (ADP-ribose) polymerase 1 (PARP1) to the damaged site to activate the DNA repair process through poly-ADP-ribosylation (PARylation) of itself and its target proteins. This process destabilizes chromatin structure and thus facilitates the access of DNA repair machinery to DNA-damaged sites (20). PARP uses nicotinamide adenine dinucleotide (NAD) as a substrate to synthesize poly(ADP-ribose; PAR) on itself and the target sites (21). PARP formation/activation is implicated in the maintenance of genomic stability, transcriptional regulation, energy metabolism, and cell death (22).

Altering PARP1 activity may prevent DNA repair resulting in the accumulation of damaged cells that leads to the activation of cell death signaling pathways (22). PARP-1 can also form a complex with the transcription factor Snail to regulate the activity of the latter factor and the levels of the intermediary filament vimentin thus affecting phenotype switching (EMT-like) and melanoma progression (23). All these features increased interest in PARP inhibitors to impede melanoma invasion and metastases.

On the other hand, IR can stimulate RTK activity and, as such, participate to promote the survival/proliferation of tumor cells. Also, it was reported that IR favors RTK activation through different mechanisms (24). In addition, IR induces intracellular translocation of RTKs, which regulates response to DNA damage activating proteins involved in DNA repair mechanisms (24), particularly the PARP-1 enzymatic activity (25, 26). Consequently, RTK-specific inhibition may impede the repair kinetics of radiation-induced DNA damage and thus improve tumor sensitivity to IR (27). This approach is supported by the successful use of MAPK inhibitors as radiosensitizers in advanced melanoma including brain metastases but the associated mechanism remains to be uncovered (28, 29).

Among various RTKs altered in melanoma, in this study we focused on c-Met signaling based on several observations suggesting its role in DNA repair in response to RT-induced DNA damage (30). Interestingly, inhibition of c-Met conferred radiosensitization in glioma, glioblastoma, NSCLC, prostate cancer cells, and their xenografts through cell cycle arrest, inhibition of DNA repair, or induction of apoptosis (31–33). Similarly, esophageal carcinoma cells (ECA109 and TE13) also showed radiosensitization by foretinib (c-Met inhibitor) (34). Despite the evidence of its role in DNA repair in specific tumors, the mechanism(s) by which c-Met signaling stimulates radiation-induced DNA repair has not been fully studied. In contrast, in a panel of five NSCLC cell lines (A549, H460, H3122, H2228, and H1993), crizotinib (c-Met inhibitor), did not significantly mediate cellular radiosensitivity, or altered DNA repair kinetics and even cell cycle distribution; besides it was noted that no consequent delay in tumor growth was observed in response to crizotinib and radiation combination (35). Therefore, understanding the mechanistic of radioresistance exhibited by c-Met in each cancer type is of particular importance for therapeutic combinations to achieve optimal radiosensitization.

To this aim, we investigated the mechanism of radioresistance conferred by c-Met in melanoma based on previous work indicating that some RTKs could activate PARP1 enzymatic activity by distinct mechanisms which are cancer dependent (25, 26). Most importantly, we found that c-Meti and/or PARPi combined with radiotherapy have a synergistic effect on WTBRAF melanoma growth inhibition both *in vitro* and *in vivo*.

## 2. Materials and methods

### 2.1. Inhibitors

Crizotinib (PF-02341066) and Olaparib (AZD2281; Selleckchem) were dissolved in DMSO according to the manufacturer’s recommendations. Stock aliquots of 10 − 2 M were stored at −20°C until use.

### 2.2. Melanoma cell lines

Human melanoma cell lines used in this study were all established in our Laboratory, from skin or lymph node metastases (36, 37). Of note, all were obtained from patients following the declaration of Helsinki and with Good Clinical Practice guidelines as defined by the International Conference on Harmonization. All patients provided written and signed informed consent before enrolment. All cell lines in this study are continuous cell lines with more than 100 passages and were regularly checked for mycoplasma contamination using MycoAlert® Mycoplasma Detection Kit (Lonza, Rockland, ME, USA). Cell line authentication was evaluated by STR method as described previously (38). BRAF, NRAS, KIT and MET mutations were assessed with the next-generation DNA sequencing for 48 genes from the cancer panel (TruSeq Amplicon—Cancer Panel, Illumina, San Diego, CA, USA), and summarized in Table 1.

**Table 1:** Gene alterations of the melanoma cell lines used in this study.

Cell line	BRAF	NRAS	c-Kit	c-Met
HBL (MM001)	WT	WT	D820Y	WT
LND1 (MM011)	WT	Q61R	Amp c-Kit	WT
MM074	V600E	WT	WT	WT
MM050	V600E	WT	WT	WT
MM162	WT	WT	WT	WT
MM094	V600E	WT	WT	WT
MM165	WT	Q61R	WT	WT

WT, wild-type; Amp, amplification.

### 2.3. Cell culture conditions

Cells were grown in HAM-F10 medium supplemented with 5% heat-inactivated fetal calf serum, 5% heat-inactivated new-born calf serum, L-glutamine, penicillin, and streptomycin at standard concentrations (all from Gibco, Invitrogen, UK) at 37°C in a humidified 95% air and 5% CO2 atmosphere. For routine maintenance, cells were propagated in flasks, harvested by trypsinization (0.05% trypsin–EDTA; Gibco), and subcultured twice weekly.

### 2.4. Cell irradiation

Cells were irradiated in a 6MV photon beam at a dose rate of about 4Gy/minon a Clinac 600 linear accelerator (Varian Medical Systems, Palo Alto, CA, USA). The collimator opening was set to 40 × 40 cm^2^, which allows the possibility of irradiating several plates at the same time. For adequate backscattering conditions, plates were placed on a 5 cm-thick polystyrene phantom. To achieve dose homogeneity and ensure electronic equilibrium, a 6 mm-thick polystyrene build-up slab covers the top of the plates.

### 2.5. Clonogenic assay

Cell survival was measured by clonogenic assay, according to Franken et al. (39). Cells were seeded on day 0, at a cell density of 1,500–2,500, depending on the line, in triplicates, in six-well plates, and incubated under regular culture conditions. On days 1, 4, 8, and 11, cells were treated with/without effectors and subsequently incubated under regular culture conditions. On day 2, cells were irradiated. On day 14, cells were fixed and stained with 6% glutaraldehyde and 0.05% crystal violet. Colonies of at least 50 cells were counted using ImageJ software. The survival fraction was calculated relative to untreated samples.

### 2.6. Cell death determination

Cells were seeded in six-well plates (2 × 10^5^ cells/well) in a culture medium. On days 1 and 4, the culture medium was replaced by a fresh one containing effectors or not. Cells were irradiated on day 2 and incubated for six additional days. Then, the supernatant was collected and adherent cells were harvested by trypsinization and returned to the previously collected medium. Cells were pelleted by brief centrifugation (200 g, 5 min) and suspended in 100 μl 1× Binding Buffer (BD Pharmingen). After the addition of 5 μl annexin V-PE and 5 μl 7-amino-actinomycin (7-AAD), cell suspensions were incubated for 15 min at room temperature and in the dark. Finally, cells were diluted with 400 μl Binding Buffer and analyzed within 1 h in a flow cytometer (FACS Calibur, Becton Dickinson, Franklin Lakes, NJ, USA).

### 2.7. Western blotting

Cells were plated in 160 cm2 Petri dishes (1.5 × 106 cells/dish) in the culture medium. Cells were lysed using a detergent cocktail (M-PER mammalian extraction buffer) supplemented with protease inhibitors (Halt protease inhibitor cocktail) and phosphatase inhibitors (Halt phosphatase inhibitor cocktail; all from Pierce, Rockford). Protein concentration was measured by the BCA Protein Assay (Pierce™ BCA Protein Assay Kit, Thermo Scientific™) using bovine serum albumin as the standard. Immunodetections used antibodies raised against, phospho-Met (1/1,000), lamin B2 (1/1,000; all from Cell Signaling Technology), PARP-1 (1/150; from Thermofisher Scientific, Life Technologies Europe BV), PAR (1/1,000; R&D systems, Bio-Techne), and β-actin (1/5,000; from Millipore, Temecula). Peroxidase-labeled anti-rabbit IgG antibody (1/5,000) or peroxidase-Duolink® PLA Flow Cytometry antibody (1/5,000; both from GE Healthcare Europe G mbH) were used as secondary antibodies. Bound peroxidase activity was revealed using the SuperSignal® West Pico Chemiluminescent Substrate (Pierce). Relative protein expressions (fold-change) were calculated and normalized relative to β-actin the whole uncropped images of the original western blots from which figures have been derived are submitted in Supplementary materials (Figures S3–S6).

### 2.8. Immunofluorescence

Approximately 20,000 cells were seeded onto coverslips, 2 days before cell irradiation. Two hours after RT, the medium was discarded and the cells were incubated with fixation reagents (formaldehyde for 10 min at 37°C and ice-cold methanol for 5 min at −20°C) and permeabilized with Triton-X, before blocking with 2% BSA for 60 min. Cells were then incubated with primary antibodies at 4°C overnight. On the second day, cells were incubated with a secondary antibody (goat anti-rabbit) for 1 h at room temperature. Following washes (three times with PBS), cells were stained with diamidino-2-phenylindole (DAPI) for 10 min and washed three times with PBS and distilled water and mounted on coverslips. Fluorescence was examined with a confocal microscope (Nikon Ti2, France).

### 2.9. CRISPR/Cas9 knockout

Cells were seeded (4 × 105 cells/well) in six-well plates 24 h before transfection, co-transfected with c-Met/PARP CRISPR/Cas9 KO or control CRISPR/Cas9 and c-Met/PARP HDR plasmids using UltraCruz transfection reagent according to the manufacturer’s protocol (CRISPR KO Transfection Protocol, Santa Cruz Biotechnologies). Transfection efficiency was monitored by red fluorescence microscopy. Transfected cells were selected by adding the puromycin antibiotic to the culture medium at 2 μg/ml according to the manufacturer’s protocol (Santa Cruz Biotechnologies). c-Met/PARP KO cells were then evaluated by Western blotting.

### 2.10. Duolink^®^ PLA flow cytometry

Cells were seeded (4.10^5^cells/well) in six-well plates. On day 1, the culture medium was replaced by a fresh one. On day 2, cells were irradiated and further incubated for 2 h. The supernatant was removed and attached cells were harvested by trypsinization, suspended in a culture medium, and prepared for flow cytometry analysis. Briefly, cells were fixed (formaldehyde for 10 min at 37°C), permeabilized (with Triton-X), and incubated overnight with primary antibodies. On the following day, cells were washed and incubated with a pair of oligonucleotide-labeled anti-rabbit IgG or anti-mouse IgG antibodies (PLA probes/Plus, Minus) to detect the corresponding primary antibodies. The addition of ligase binds the oligo-labeled proteins of interest if they were in immediate proximity. Then the signal is amplified using polymerase followed by cell wash, incubation in a detection solution and analyzed within 1 h in a flow cytometer (FACS Calibur, Becton Dickinson, Franklin Lakes, NJ, USA).

### 2.11. Subcellular fractionation

Cells were plated in Petri dishes (1.5 × 106 cells/dish) in culture medium. One day after plating, the culture medium was replaced by a fresh one containing or not effectors. On day 2, cells were irradiated and incubated for 2 h. Cells were washed and harvested by trypsinization. The cell pellet was resuspended in a pre-extraction buffer for 10 min on ice and then centrifuged for 1 min at 6,000 g which allows extraction of the cytoplasmic fraction. The remaining cell pellet was suspended in an extraction buffer for 15 min on ice and the nuclear fraction was collected by centrifugation for 10 min at 8,000 g and 4°C according to the manufacturer’s protocol (ab113474 – Nuclear Extraction Kit).

### 2.12. Phosphorylated H2AX

Phosphorylated H2AX level was evaluated using a fluorescent specific antibody and subsequent evaluation in a flow cytometer as follows: One day after plating cells in 6-well plates (3 × 105 cells/well), the culture medium was replaced by a fresh one, containing or not effector, and cells were irradiated 1 day later. Two hours after RT, cells were fixed, permeabilized, and phosphorylated H2AX level was measured by DNA Damage Kit (BD Pharmingen, Erembodegem-Dorp, Belgium), according to the manufacturer’s recommendations.

### 2.13. Human melanoma xenografts and mice irradiation

Five to six-week-old female nude (nu/nu) mice weighing 17–21 g were purchased from Charles River Laboratories (Saint Aubin lès Elbeuf, France). Mice were subcutaneously injected (right and left leg) with 2.5 × 106 HBL cells in 150 μl of 50% Matrigel (from Trevigen, Gaithersburg, MD, USA) in saline solution. When tumors reached about 200 mm3, mice were randomized into four groups of 5 mice each and intraperitoneally injected daily (5 days) with vehicle (DMSO), 50 mg/kg of Olaparib, and/or 5 mg/kg of Crizotinib.

The right leg was irradiated (2Gy) on a Clinac 600 on days 1, 2, 4, and 5. The mice were placed in an in-house developed phantom based on a 12 mm-thick polystyrene plate, 3D-printed polylactic acid (PLA) pillars glued to the base plate to separate the mice from each other, and an 8 mm polystyrene build-up cover plate. The setup allowed irradiating 5 mice at the same time, as shown in Supplementary Figure 1.

The legs were irradiated with anterior and posterior 6MV photon beams to ensure homogeneous dose coverage on the target and asymmetrical field sizes to shield out the rest of the body.

Tumor size and body weight were measured every 3 days. Tumor volumes were calculated using the formula (L × W × W)/2, in which L is the length and W is the width, as measured with a Vernier caliper. Immediately after dissection, tumor xenografts were fixed and embedded in paraffin. The experiments were performed in accordance with the European Union Guidelines and validated by the local Animal Ethics Evaluation Committee “Comité d’éthique du Bien-Etre Animal-Université Libre de Bruxelles” (CEBEA) protocol: 746 N.

### 2.14. Immunohistochemistry staining

After dissection, tumors were immediately fixed in 10% formalin, transferred when appropriate to 70% ethanol solution and stored at 4°C. Samples were embedded in paraffin, and 4-μm sections were prepared for immunostaining with hematoxylin and eosin (HE), Ki67 (3 μg/ml; R&D systems, Bio-Techne), and cleaved caspase (1/50; Thermo Fisher Scientific). Stained sections were imaged using an NDP Slice Scanner (Hamamatsu, Hamamatsu City, Japan). Five regions were selected at random on different parts of the section and analyzed at 15x magnification, using ImmunoMembrane and ImmunoRatio web applications.

### 2.15. Statistical analysis

All data were calculated using GraphPad Prism software (GraphPad Software, La Jolla, CA, USA). Data are expressed as means ± SEM of at least three independent experiments. Significance *p < 0.05, **p < 0.01, and ***p < 0.001 were calculated by Student’s *t*-test and two-way Analysis of Variance (ANOVA) and *post hoc* tests.

### 2.16. Combination index calculation

The combined effect was analyzed by the multiple drug-effect equations and quantified by the combination index (CI) using CompuSyn software. The Chou-Talalay method for combination evaluation is established on the median-effect equation, which gives the theoretical basis for the combination index (CI)-isobologram that favors quantitative evaluation of drug–drug, drug-RT, drug–drug-RT interactions, where CI <1, =1, and >1 indicate synergism, additive, and antagonism effect, respectively.

## 3. Results

### 3.1. Functional role of PARP-1 in melanoma radioresistance

**Figure 1:**
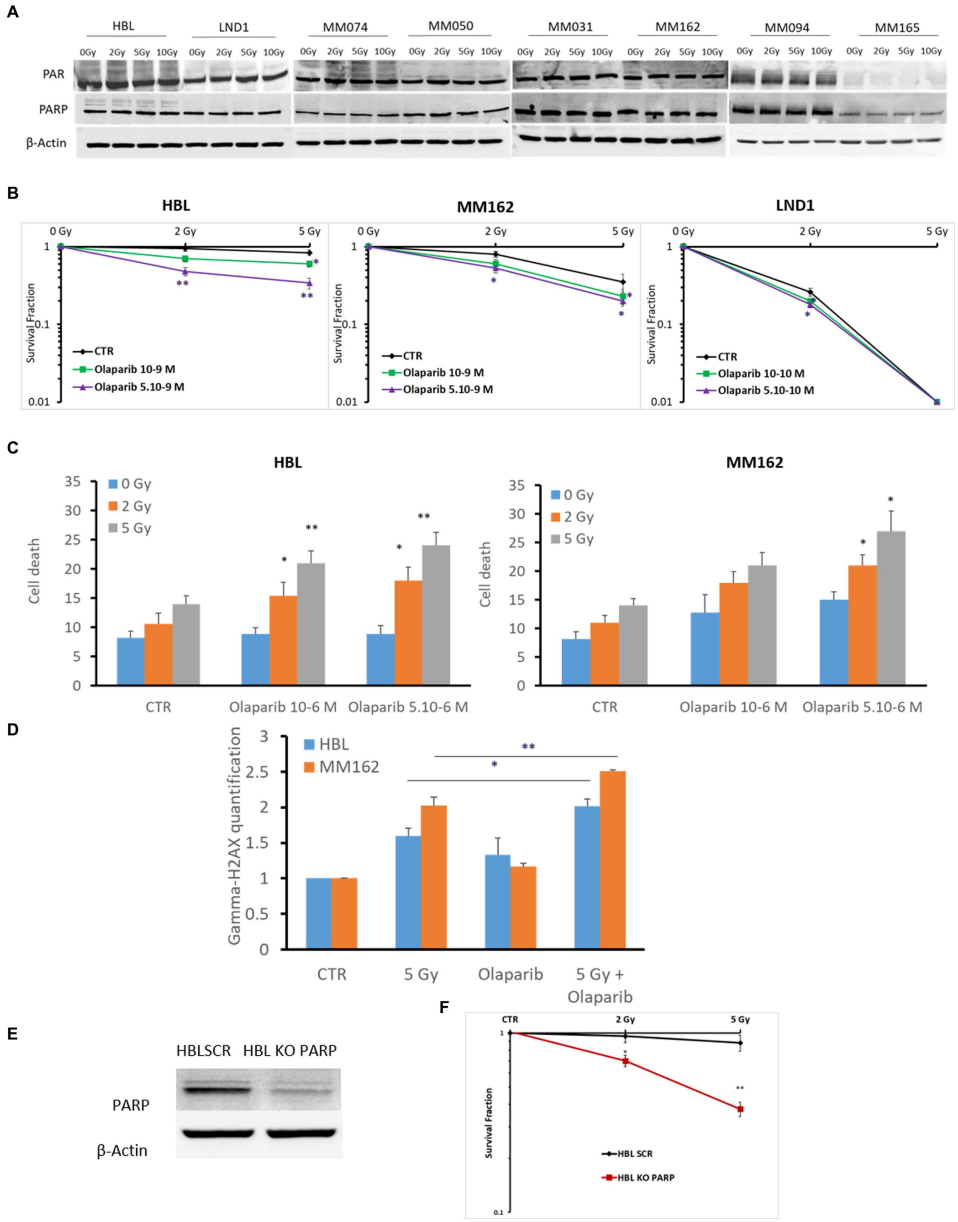
Evaluation of PARP-1 expression and role in melanoma radioresistance. **(A)** Western blot showing expression of PAR, PARP-1, and β-Actin in lysates of the indicated human melanoma cell lines (one representative blot shown out of 3 replicates with the same profile), 2 h following exposure to RT. **(B)** Clonogenic survival assay of human melanoma cell lines with different irradiation doses alone or in combination with Olaparib. Surviving fractions were calculated relative to plating efficiencies. **(C)** Melanoma cells were treated with Olaparib on days 1 and 4, irradiated on day 2, and incubated for six additional days. Cell death was assessed by apoptosis (annexin-V positive cells) + necrosis (7-AAD positive cells) analysis for irradiated and non-irradiated cells treated with Olaparib (10–6; 5.10-6 M). **(D)** Melanoma cells were treated with Olaparib (10–6 M), 24 h before exposure to RT. Then DNA damage was assessed by γ-H2AX (mean fluorescence intensity) 2 h after RT (*n* = 3). Data are presented as means ± SEM of at least three independent experiments. Gy, Gray; CTR: untreated control. **(E)** Western blot showing KO of PARP in HBL melanoma cells. **(F)** clonogenic cell survival assay of irradiated HBL SCR and HBL KO PARP-1. Quantification of surviving fractions from at least three independent experiments is shown. Data are presented as means ± SEM (*n* = 3) compared to HBL SCR cells, *p < 0.5; **p < 0.01; (Student’s *t*-test); SEM, standard error of the mean.

PARP is a component of the BER complex (40). PARP is a 1,014 amino acid protein (~116 kDa), which exhibits an intrinsic ADP-ribosyltransferase activity that transfers ADP-ribose from donor NAD+ molecules to glutamate. Particularly, PARP mediates SSBR which leads to decreased damage by RT. Therefore, in theory, radioresistance could be associated with the high activity of PARP in melanoma. To address this question, we investigated the constitutive level of PARP-1 and its activity in different melanoma lines harboring various gene mutations under increasing doses of RT as it has not been reported yet in melanoma. We observed a high basal level and activity revealed by PAR expression using PAR/pADPr antibody. Of note, this antibody is specific for PAR polymers 2–50 units long, which could be formed following PARP activation which mediates the increase in the synthesis of PAR polymer on PARP in almost all melanoma lines (*n* = 8), except MM165. Melanoma cells conserved high PARP-1 expression and activity under any IR dose. This indicates a high PAR enzymatic activity and expression in melanoma not significantly affected by IR (Figure 1A). Secondly, and to evaluate the possible role of PARP-1 in melanoma radioresistance, we investigated the benefit of inhibiting PARP-1 in three melanoma cells (2 radioresistant: HBL and MM162, and one radiosensitive: LND1). Indeed, Olaparib in combination with increasing doses of RT significantly decreases the survival fraction in all three lines (Figure 1B). Of note, Olaparib is used in two non or slightly toxic concentrations (Supplementary Figures S2A–C) which correspond to IC5 & IC10 for HBL, and IC15 & IC20 for both MM162, and LND1. In addition, the effect on cell survival is accompanied by an increase in both cell death (Figure 1C) and DNA damage as revealed by γH2AX phosphorylation (Figure 1D). Furthermore, and to validate the role of PARP-1 in melanoma radioresistance, we performed a specific knock-out (KO) of the PARP-1 gene in the radioresistant HBL cells (Figure 1E) that caused a pronounced decrease in cell survival after exposure to RT compared to HBL SCR (Figure 1F), supporting the functional role of PARP-1 in melanoma radioresistance.

### 3.2. Targeting c-met enhances response to RT in melanoma

Compelling evidence suggests that aberrant RTK signaling in many tumors can moderate RT efficacy mainly through direct activation of DNA repair machinery thus contributing to tumor radioresistance. However, the underlying exact molecular mechanisms remain to be uncovered.

**Figure 2:**
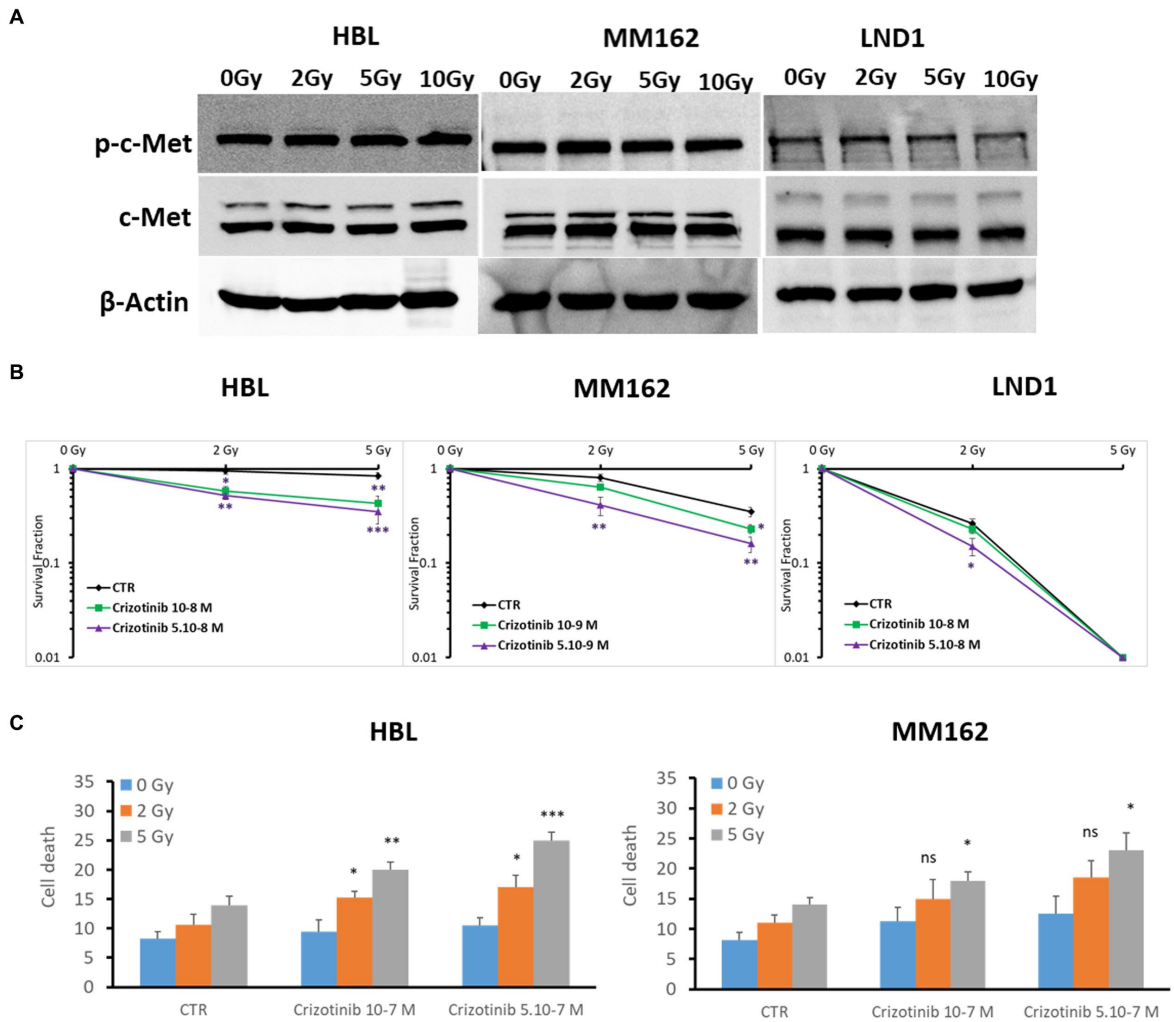
c-Met expression and its association with melanoma radioresistance. **(A)** Western blot showing expression and phosphorylation of c-Met in lysates of human melanoma cell lines (one representative blot shown out of 3 replicates with the same profile), 2 h after RT. **(B)** Clonogenic survival assay of human melanoma cell lines with different irradiation doses alone or in combination with Crizotinib. Surviving fractions were calculated relative to plating efficiencies. Data were presented as means ± SEM of at least three independent experiments. Gy, Gray; CTR: untreated control. **(C)** Melanoma cells were treated with Crizotinib on days 1 and 4, irradiated on day 2, and incubated for six additional days. Apoptotic cell death (annexin-V positive cells +7-AAD negative/positive cells) analysis of irradiated and non-irradiated cells treated or not with Crizotinib. Data are presented as means ± SEM (*n* = 3), *p < 0.5; **p < 0.01; ***p < 0.001 (Student’s *t*-test) compared to RT alone; SEM, standard error of the mean.

In this study, we focused on the c-Met role in melanoma radioresistance as it is often overexpressed in WTBRAF melanomas and it has been reported that HGF/c-Met Signaling contributes to several processes that are crucial for melanoma development, such as proliferation, survival, motility, and invasiveness, including distant metastatic niche formation (7). First, we found high basal but similar expression of c-Met under increasing doses of RT (Figure 2A). Then, we examined the radiosensitizing effect of the c-Met inhibitor Crizotinib in three cell lines with different radiosensitivity/resistance. Of note, Crizotinib is used at two non or slightly toxic concentrations, which correspond to IC10 and IC15 for HBL, IC10 and IC20 for MM162, and IC20 and IC30 for LND1 (Supplementary Figures S2D–F, respectively). Targeting c-Met with Crizotinib in combination with RT mediated a pronounced decrease in survival fraction which was associated with an increase in cell death (Figures 2B,C). This indicates that targeting c-Met could enhance RT response in melanoma but the underlying mechanisms are yet to be uncovered.

### 3.3. RT favors nuclear translocation of c-met and its association with PARP-1 in melanoma

To investigate the underlying molecular mechanisms regulating radioresistance in WTBRAF melanoma and identify potential targets, we searched for RTKs that are associated with PARP-1 after RT. Molecular crosstalk between c-Met and PARP-1 was reported in breast cancer and hepatocellular carcinoma to confer resistance to PARP inhibitors under oxidative stress (25). As targeting c-Met or PARP-1 affects melanoma cell survival and response to RT, we examined a possible RT-promoted interaction between c-Met and PARP-1.

**Figure 3:**
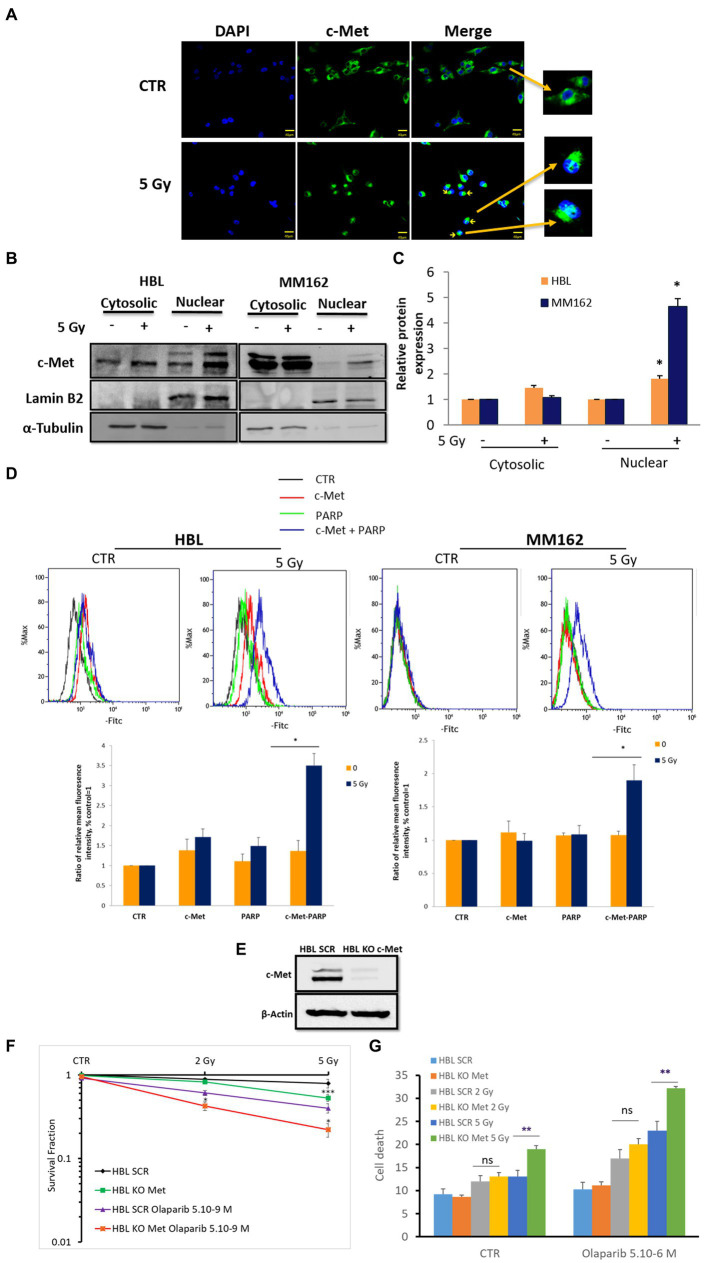
RT promotes c-Met-PARP-1 interaction in melanoma. **(A)** Sub-confluent melanoma cells were fixed and stained with C-terminal antibody for c-Met (C-28, green) and DAPI for nucleus (blue), 2 h after exposure to RT. Representative images show an RT-induced increase in c-Met intracellular translocation (fluorescent patches). **(B)** Subcellular fractions of cell lysates were analyzed by western blot. Cross-contamination was checked by a nuclear marker (Lamin B2) and a cytoplasmic marker (α-Tubulin). **(C)** The histogram shows normalized c-Met protein expression levels of control versus 5 Gy in the cytoplasm and nucleus; data are means ± SEM (*n* = 3). **(D)** Proximity ligation assay (PLA) was used to detect PARP-1 and c-Met interaction (blue signals), 2 h after exposure to 5 Gy RT. Representative images (up) and quantification of PLA signals (mean fluorescence intensity) from three independent experiments. Error bars represent SEM, *p < 0.05, *t*-test. **(E)** Western blot showing c-Met expression in c-Met-knockout cells. HBL SCR and HBL KO c-Met cells were treated with Olaparib and/or RT and subjected to **(F)** clonogenic survival assay **(G)** cell death evaluation. Data are presented as means ± SEM (*n* = 3) of HBL KO c-Met (untreated & treated Olaparib) *Vs* HBL SCR cells (untreated & treated Olaparib), *p < 0.5; **p < 0.01; ***p < 0.001 (Student’s *t*-test).

As PARP-1 is a nuclear protein, and c-Met is a membrane receptor tyrosine kinase that can be found in the nucleus under oxidative stress, we first evaluated the effect of IR on c-Met membrane versus nuclear expressions and found that while it can be most abundant on the cell membrane, it can also be found near the nucleus, (Figures 3A,B; perinuclear localization). Interestingly, RT promoted c-Met nuclear expression in both lines 2 h following exposure to RT (Figures 3A–C). This indicates the possibility of an RT-mediated c-Met/PARP-1 interaction in melanoma. To this end, we used the Duolink PLA assay to monitor such interaction and indeed we observed a significant increase in PLA signals with RT compared to non-irradiated cells in the same conditions as above indicating c-Met/PARP-1 association after RT in melanoma (Figure 3D).

### 3.4. C-met promotes PARP-1 activity under RT and confers melanoma radioresistance

To further assess the importance and the consequences of RT-promoted c-Met/PARP-1 interaction, we proceeded with three distinct approaches: (1) c-Met KO; (2) specific dual inhibition of c-Met and PARP-1 combined with RT, and (3) monitoring PARP-1 activity following c-Met inhibition and RT.

First, c-Met KO (Figure 3E), similar to its pharmacological inhibition with Crizotinib (Figure 2B), enhanced sensitivity to RT further supporting the role of c-Met in melanoma radioresistance (Figure 3F). Interestingly, Olaparib caused a more pronounced effect revealed by a decrease in cell survival fraction and an increase in cell death in HBL-KO c-Met in response to RT (5Gy) compared to HBL-SCR (Figures 3F,G) in line with the RT-promoted c-Met/PARP-1 interaction.

**Figure 4:**
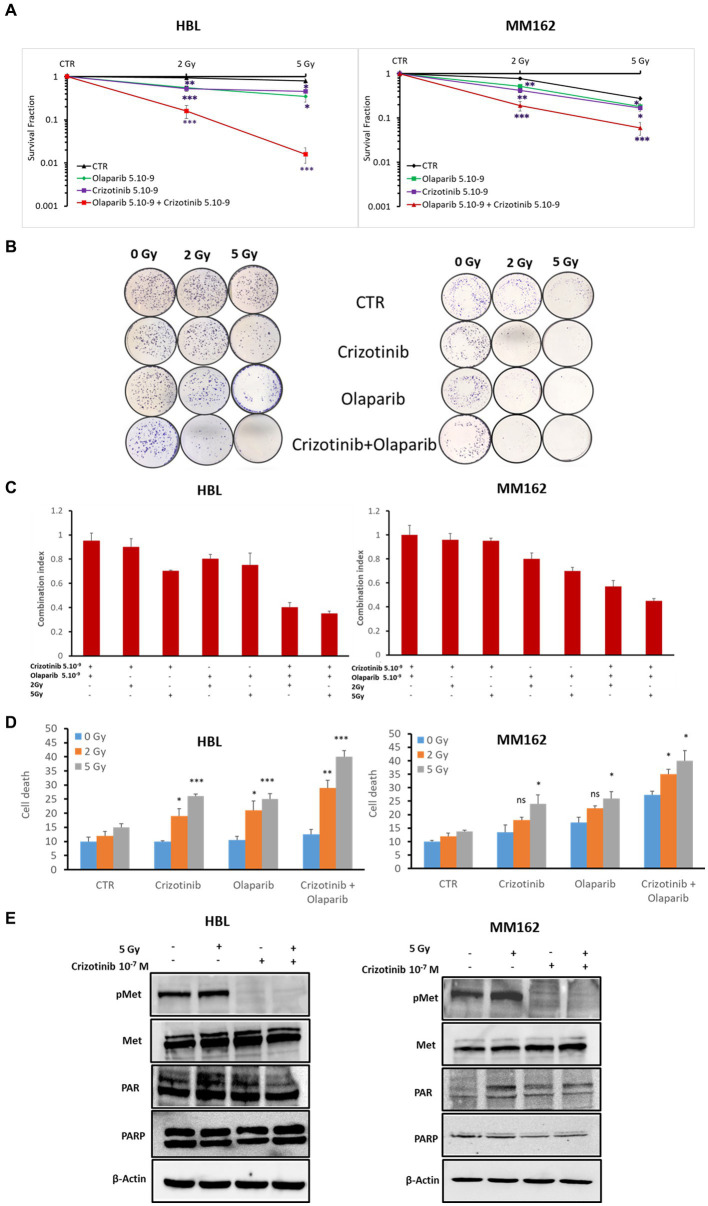
c-Met and PARP-1 interaction confer melanoma radioresistance. **(A)** Clonogenic survival assay of HBL and MM162 human melanoma irradiated either to 2 Gy or 5 Gy alone or in combination with non-toxic concentrations of Crizotinib and/or Olaparib. Surviving fractions were calculated relative to plating efficiencies. Data were presented as the mean ± SEM of at least three independent experiments. *p < 0.5; **p < 0.01; ***p < 0.001 (Student’s *t*-test) compared to RT alone. **(B)** Representative images of clonogenic assays. **(C)** The interaction between Crizotinib, Olaparib, and RT was examined using CompuSyn software where the combination index (CI). CI = 1 indicates an additive effect, CI <1, a synergism, and CI >1, an antagonism. **(D)** Measurement of cell death (apoptosis, annexin-V positive cells, and necrosis, 7-AAD positive cells). Data are presented as means ± SEM (*n* = 3) compared to non-irradiated cells, untreated or treated (Crizotinib or Olaparib or Crizotinib and Olaparib), *p < 0.5; **p < 0.01; ***p < 0.001 (Student’s *t*-test). **(E)** Western blot showing expression of c-Met, p-c-Met, PAR, and PARP-1 in lysates of human melanoma cell lines treated with 5 Gy and/or 10–7 M of Crizotinib (one representative blot shown out of 3 replicates with the same profile). β-actin was used as a loading control.

Second, to provide more evidence about the activation of PARP-1 by c-Met under RT in melanoma, we evaluated the radiosensitizing effect of the dual combination of Crizotinib and Olaparib. While the combination of RT with Crizotinib and the combination of RT with Olaparib significantly reduced cell survival in both melanoma lines (HBL, MM162) compared to RT alone (Figure 4A), it was more efficient with the dual targeting of c-Met and PARP-1 and could prevent any residual colony formation as compared to RT + Olaparib or RT + Crizotinib (Figures 4A,B). Importantly, the combination index (Figure 4C) shows that the triple combination has a strong synergistic effect by significantly reducing cell survival and favoring cell death (Figure 4D).

Third, coherently and to further provide evidence and investigation of the c-Met association and subsequent activation of PARP-1 under RT, we treated HBL and MM162 cells with Crizotinib 24 h before exposure to 5 Gy and evaluated PARP-1 activity revealed by PAR level. Indeed, 2 h cell exposure to RT alone caused an increase in PAR levels. Intriguingly, treatment with Crizotinib mediates c-Met phosphorylation inhibition and opposed RT-induced PARP-1 activity compared to RT alone (Figure 4E).

### 3.5. Co-targeting PARP-1-1 and c-met radiosensitizes WTBRAF melanoma *in vivo*

**Figure 5:**
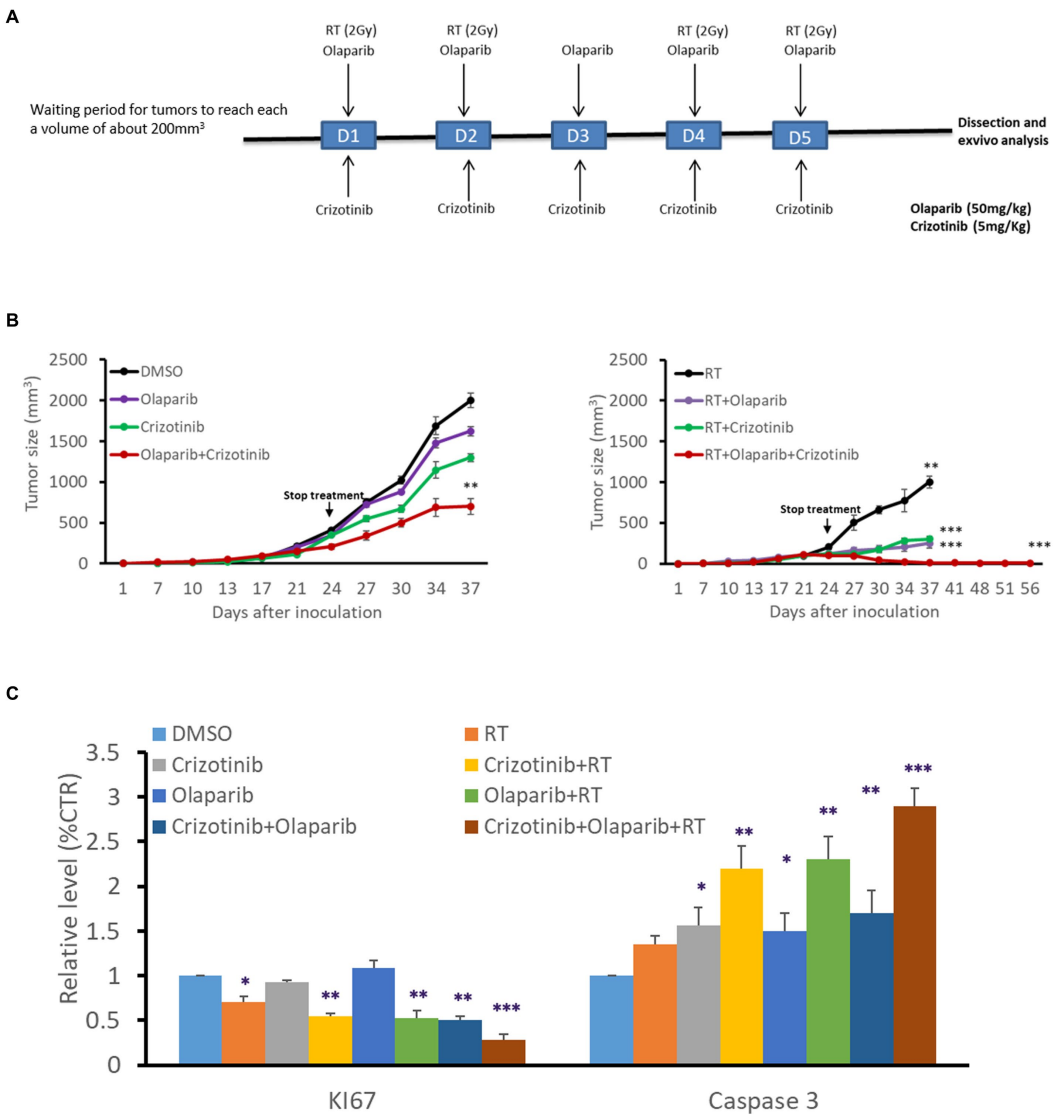
PARP-1 and c-Met inhibition enhanced melanoma radiosensitivity *in vivo*. **(A)** Swiss nude mice (nu/nu) bearing HBL melanoma on both lower limbs were treated once a day over 5 consecutive days with Crizotinib (5 mg/kg), Olaparib (50 mg/kg), or their combination. The right leg was irradiated (2 Gy) on days 1,2,4 and 5, 30 min after treatment with vehicle or Crizotinib and/or Olaparib. **(B)** Tumor volume of non-irradiated (left panel), irradiated (right panel), untreated, or treated mice with drugs, in the period between D0 and D56. *p < 0.05, **p < 0.01, ***p < 0.001 (two-way ANOVA) compared to non-irradiated. **(C)** Relative expression (IHC) of Ki67 and cleaved caspase 3 as compared to respective controls. Data are presented as mean ± SEM.

We evaluated the benefit of PARP-1 and c-Met dual inhibition with RT *in vivo* in a melanoma xenograft model (Figure 5) using the radioresistant HBL melanoma cell line. Tumor cells were subcutaneously injected into both lower legs and tumor growth was monitored to reach a volume of about 200 mm3 before intraperitoneal administration of the effectors (5 mg/kg Crizotinib and/or 50 mg/kg Olaparib, for 5 days). On days 1–2–4–5, and 30 min after drug administration, the mice were exposed to a single-dose RT (2 Gy) delivered specifically to the right leg, while protecting the rest of the body with a lead shield (Figure 5A). RT alone caused a decrease in tumor growth compared to non-irradiated (left panel) while drug combination substantially inhibited tumor growth compared to either inhibitor alone (Figure 5B). Interestingly, the combination of RT either with c-Met or PARP-1 inhibition showed a more pronounced inhibitory effect on tumor growth in comparison with non-irradiated tumors (left panel). More interestingly, the triple combination of RT, Olaparib, and Crizotinib resulted in significantly better tumor control (Figure 5B). The latter was strongly supported by the finding that tumor regrowth did not occur in irradiated tumors as monitored for about 8 weeks after the stop of all treatments and by examining tumor tissues after their removal in terms of proliferation (Ki67 staining) and increased cell death (cleaved caspase 3; Figure 5C). Of note, none of the treatment regimens affected animal weight monitored at each tumor size measurement reflecting a low, if any, general toxicity. In addition, no change was observed in animal behavior, skin irritation, fever or muscle affection, confiming safety of the combination treatment.

## 4. Discussion

Currently, there is no effective treatment for WTBRAF melanoma, which represents 40–50% of all melanoma patients (41). Several trials evaluating immunotherapy PDL1/PD1 combined with other therapies are in progress for this group of patients(NCT03484923). Combinations include chemotherapy, MAPK inhibition, tyrosine kinase inhibition, or other modalities (42) underlining the need for further preclinical and clinical studies that could lead to novel innovative treatment approaches in this subgroup (2).

Although melanoma is a radioresistant tumor, radiotherapy is considered for its management in advanced stages. For example, radiotherapy is used in adjuvant settings for patients suffering from distant metastases as well as after surgery for those with a predicted high risk for recurrence such as mucosal melanoma. These are rare as compared to cutaneous melanomas but are considered a clinically aggressive subgroup of high local recurrence following surgery (43, 44). RT as an adjuvant treatment used in this group following surgery showed a remarkable tumor control benefit (45, 46). Likewise, radiosurgery is used with some success in curative settings for brain metastases, and stereotactic body RT achieves tumor control in oligometastatic melanoma with only limited toxicity. Therefore, considering the important recent advances in immunotherapy and targeted drugs, a deep understanding of melanoma mechanisms of radioresistance is essential to provide efficient combinations with effective radiosensitizing properties able to break such radioresistance.

In melanoma, several radioresistance mechanisms and associated signaling pathways were described, particularly in the case of constitutive activation of the MAPK pathway in a p53 inactivation background (29).

One of the general mechanisms of radioresistance is mediated by receptor tyrosine kinases in several malignancies mainly through interaction with DNA repair proteins. In melanoma, these are altered at different stages and can be associated with radioresistance, particularly in the mucosal subtype. However, RTK interaction with DNA repair proteins is barely studied in melanoma and only a few reports discussed its association with radioresistance.

On the other hand, PARP-1 inhibitors have been tested and evaluated in several tumor types (47, 48). Several recent studies demonstrated the clinical benefit of targeting PARP-1 with Olaparib in advanced prostate cancer harboring defects in DNA repair (49). Such benefit appears to be strongest in patients with BRCA mutations or with other defects in homologous recombination DNA repair. The same efficacy was also demonstrated in other cancer types (50).

As radioresistance could be associated with high repair capacities that prevent accumulation of DNA damage, we studied PARP-1 expression, activity, and its possible involvement in melanoma radioresistance in a panel of WTBRAF melanoma cell lines harboring different gene alterations. First, we checked and found a relatively high constitutive expression and activity of PARP-1 in all melanoma lines. Second, we verified that PARP-1 inhibition does not significantly affect cell growth in all of the lines we tested. Third, as melanoma radioresistance can mainly be due to its DNA repair efficiency, we investigated the benefit of inhibiting PARP-1 to oppose such resistance in WTBRAF melanoma. Indeed, the combination of radiotherapy and a PARP inhibitor (Olaparib) significantly decreased cell survival by increasing DNA damage and cell death in accordance with recent reports describing a sensitizing effect of the PARP inhibitors Talazoparib and Niraparib in melanoma as well (51).

However, while we observed a clear radiosensitizing effect of Olaparib, the latter was quite variable among the cell lines, also as suggested in other reports (43). Furthermore, we added substantial proof for PARP-1 role in melanoma radioresistance by performing a KO of the gene, leading again to a significant radiosensitizing effect.

Two observations paved the way to further investigate the radioresistance mechanism linked to RTKs: (1) RTKs, particularly, c-Met and EGFR, have been associated with radiation resistance in several malignancies such as HNSCC, NSCLC, and GBM (52, 53); (2) preclinical data indicated that radioresistance due RTK activation was mediated by DNA repair protein activation or by apoptosis inhibition (24, 27).

Indeed, we observed high basal and stable expression of phosphor-c-Met in all lines tested under RT. Interestingly, c-Met stands out from the latter RTKs due to its ability to translocate to the nucleus and possibly interact with PARP-1 to stimulate its activity. So, RT appears to act as a double-edged sword, generating free radicals to damage DNA but at the same time promoting its repair.

Several consecutive findings of our work support a c-Met role in melanoma radioresistance through its association and activation of PARP-1: (1) c-Met specific inhibition or KO alike radiosensitizes melanoma cells by affecting cell proliferation and increasing cell death; (2) RT promotes c-Met translocation to the perinuclear envelop and its association with PARP-1; (3) dual targeting of c-Met and PARP-1 with RT resulted in a significant decrease in cell viability and increase in cell death; (4) The triple combination of c-Met/PARP-1 inhibitions and ionizing radiation caused a long term melanoma tumor control without evidence of regrowth only in those irradiated in a xenograft animal model.

Thus, in WTBRAF melanoma, c-Met mediates the activation of PARP-1 upon exposure to ionizing radiations in a similar way described for breast cancer and hepatocellular carcinoma under oxidative stress. But unlike HCC (47, 54), melanoma shows no evidence for any RT-induced EGFR/c-Met heterodimer formation to mediate PARP-1 activity (data not shown).

Considering that most WTBRAF melanoma tumors show alterations in c-Met expression with at least 24% of receptor amplification, it can be reasonably speculated that a common subset of melanoma patients can be selected for the triple combination although adverse events may be an issue, and would have to be carefully monitored.

## Data availability statement

The datasets presented in this study can be found in online repositories. The names of the repository/repositories and accession number(s) can be found in the article/Supplementary material.

## Ethics statement

The animal study was reviewed and approved by the local Animal Ethics Evaluation Committee “Comité d’éthique du Bien-Etre Animal-Université Libre de Bruxelles” (CEBEA) protocol: 746 N.

## Author contributions

MS: conceptualization, data curation, formal analysis, methodology, and roles/writing—original draft. AN: investigation, methodology, project administration, supervision, validation, and visualization. CV: methodology. FK: methodology. YJ: methodology, writing—review and editing. FJ: writing—review and editing, formal analysis. AA: supervision, funding acquisition, resources, conceptualization. DG: writing—review and editing, formal analysis. GG: supervision, conceptualization, funding acquisition, roles/writing—original draft, and review and editing. MK: conceptualization, funding acquisition, roles/writing—original draft, and review and editing.

## Funding

This study was supported by “L’Association Jules Bordet” and a Televie Grant No. 7651819F from the National Fund for Scientific Research (Belgium). Malak Sabbah is the recipient of a Televie Grant No. 7651819F.

## Conflict of interest

The authors declare that the research was conducted in the absence of any commercial or financial relationships that could be construed as a potential conflict of interest.

## Publisher’s note

All claims expressed in this article are solely those of the authors and do not necessarily represent those of their affiliated organizations, or those of the publisher, the editors and the reviewers. Any product that may be evaluated in this article, or claim that may be made by its manufacturer, is not guaranteed or endorsed by the publisher.
