# Supplementary material for: The benefit of co-targeting PARP-1 and c-Met on the efficacy of radiotherapy in wild type BRAF melanoma
Source: Front Med (Lausanne). 2023 May 4;10:1149918. doi: 10.3389/fmed.2023.1149918 (PMC10192576; doi:10.3389/fmed.2023.1149918)
Supplement: Supplementary file 1 [file Data_Sheet_1.DOCX]

**Supplementary Materials:**

**
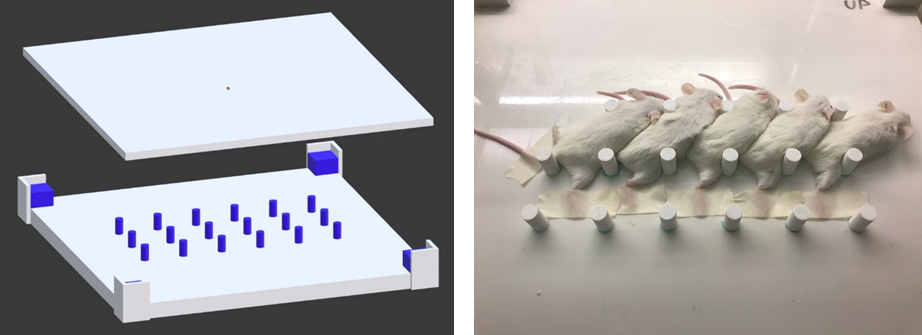
**

Figure S1: Mice irradiation setup. The legs were irradiated with anterior and posterior 6MV photon beams to ensure homogeneous dose coverage on the target and asymmetrical field sizes to shield out the rest of the body.


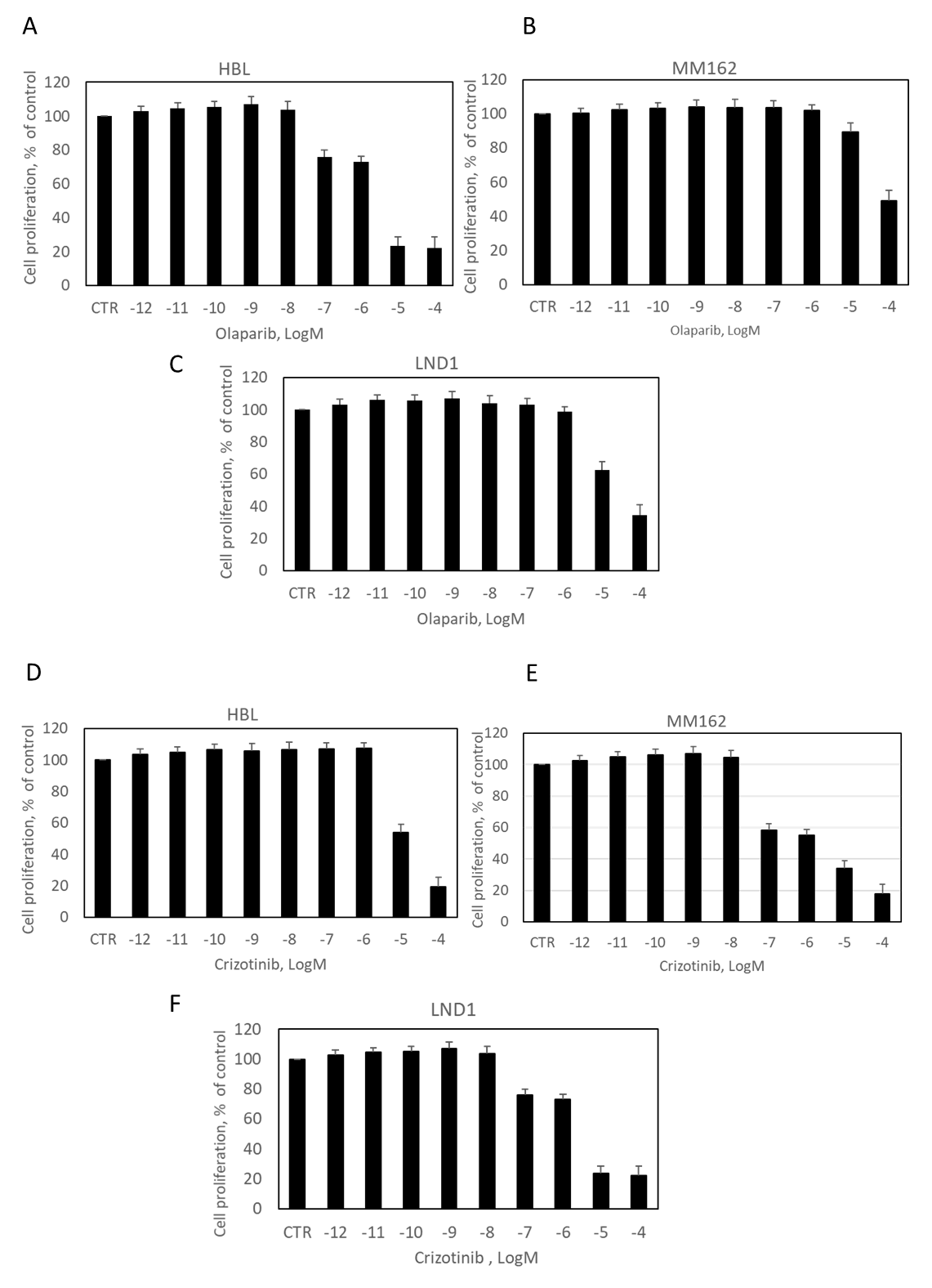


Figure S2: Characterization of melanoma cells sensitivity to Olaparib and Crizotinib. Effect of increasing concentrations of Olaparib (10-12–10-4 M) on the proliferation of (A) HBL, (B) MM162, and (C) LND1 melanoma cells, 3 days after treatment. Effect of increasing concentrations of Crizotinib (10-12–10-4 M) on the proliferation of (D) HBL, (E) MM162, and (F) LND1 melanoma cells, 3 days after treatment. Crystal violet staining. Data are presented as means ± SEM (n = 3).


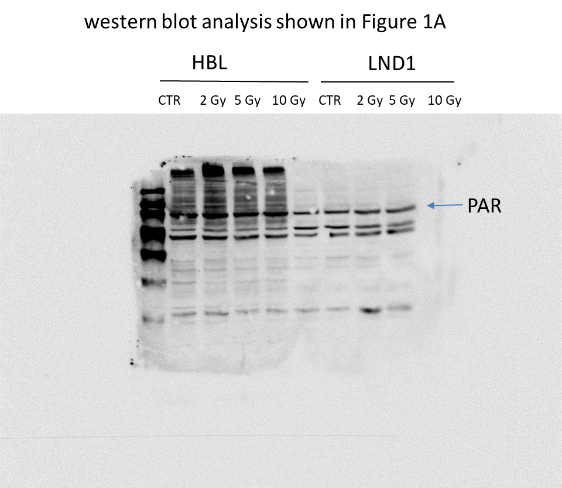

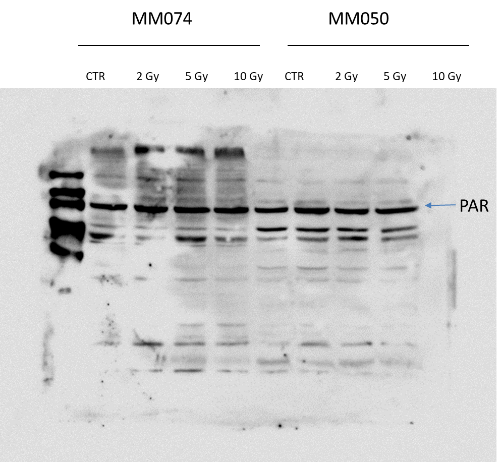


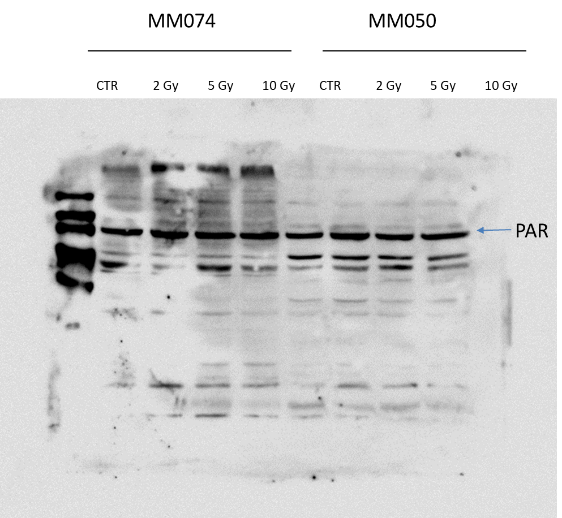

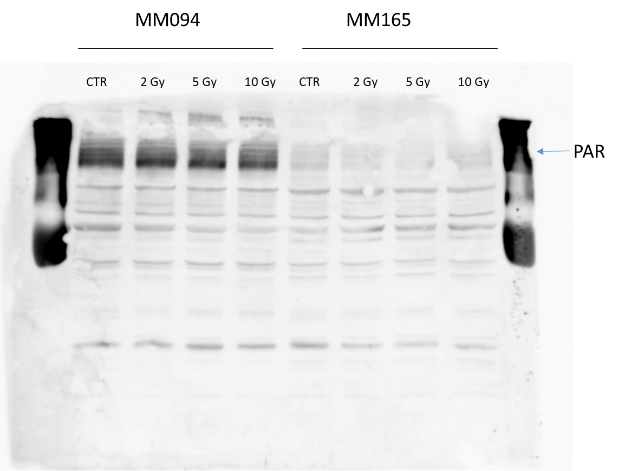


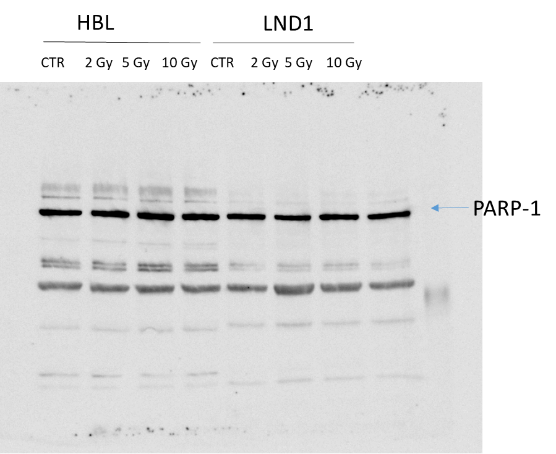

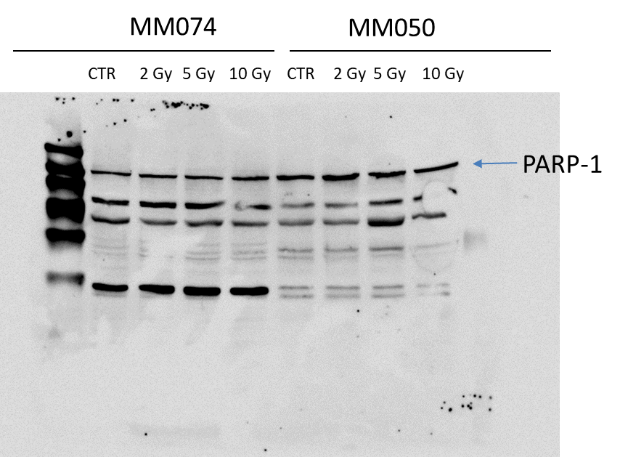


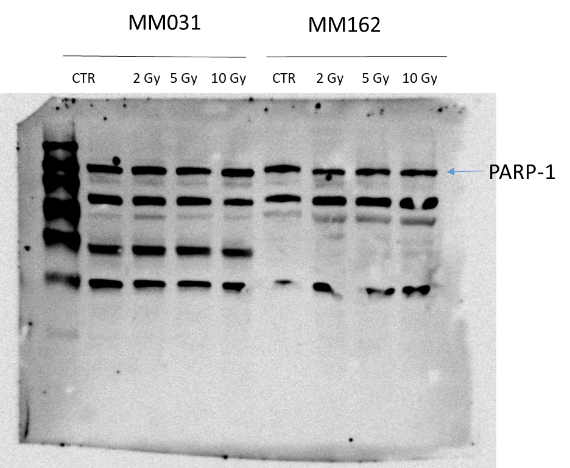

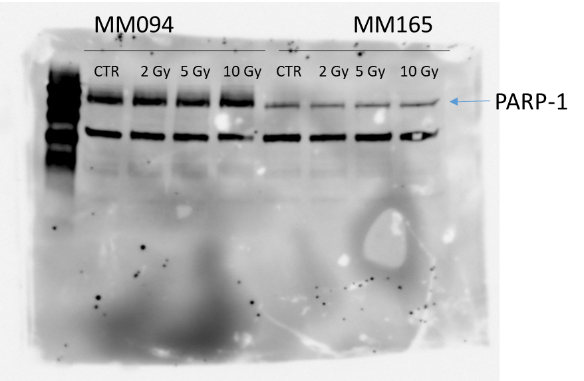


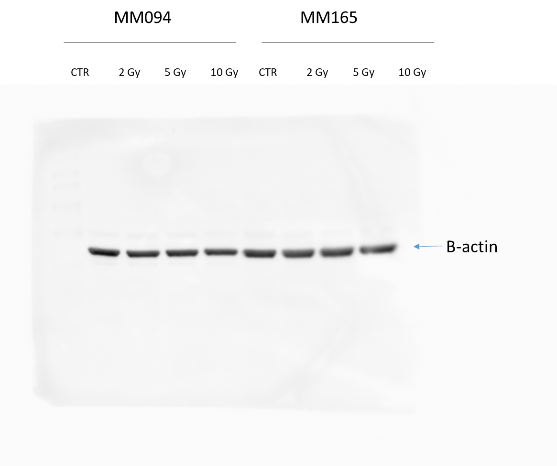

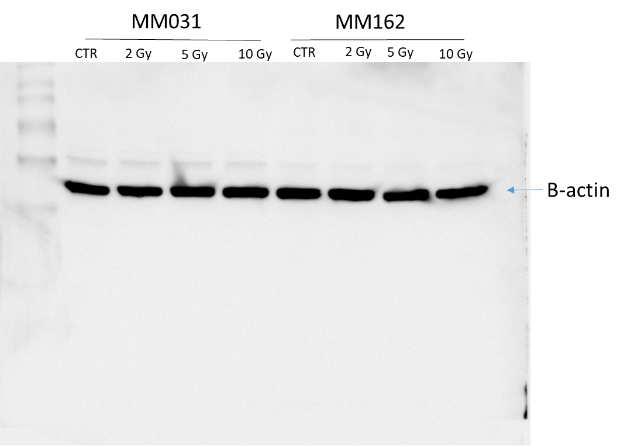


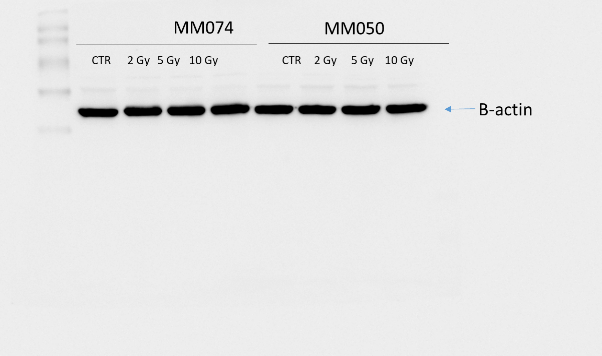

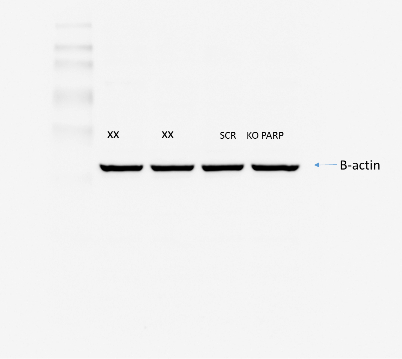


Figure S3: Uncropped images of the original western blots from western blot analysis shown in Figure 1A-1E.


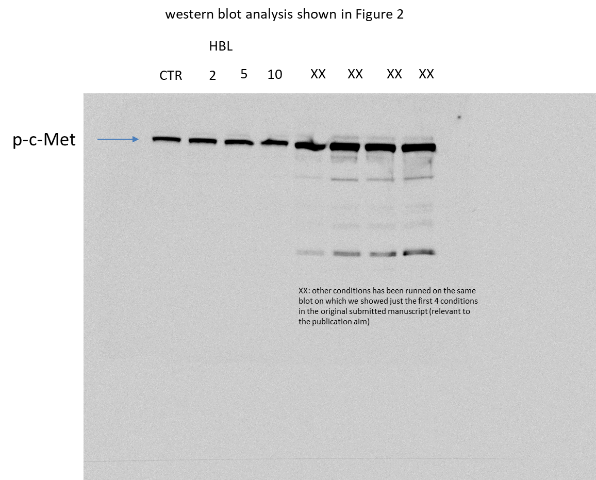

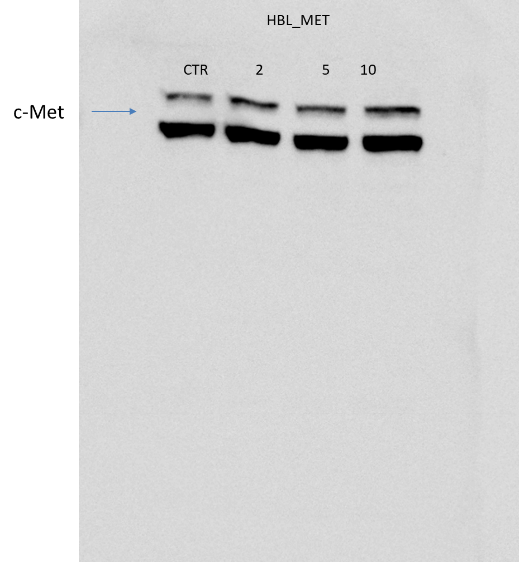


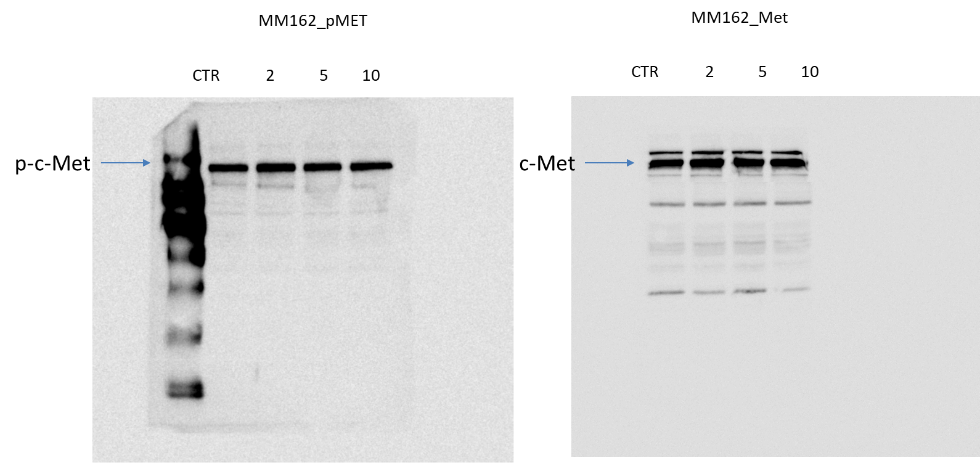


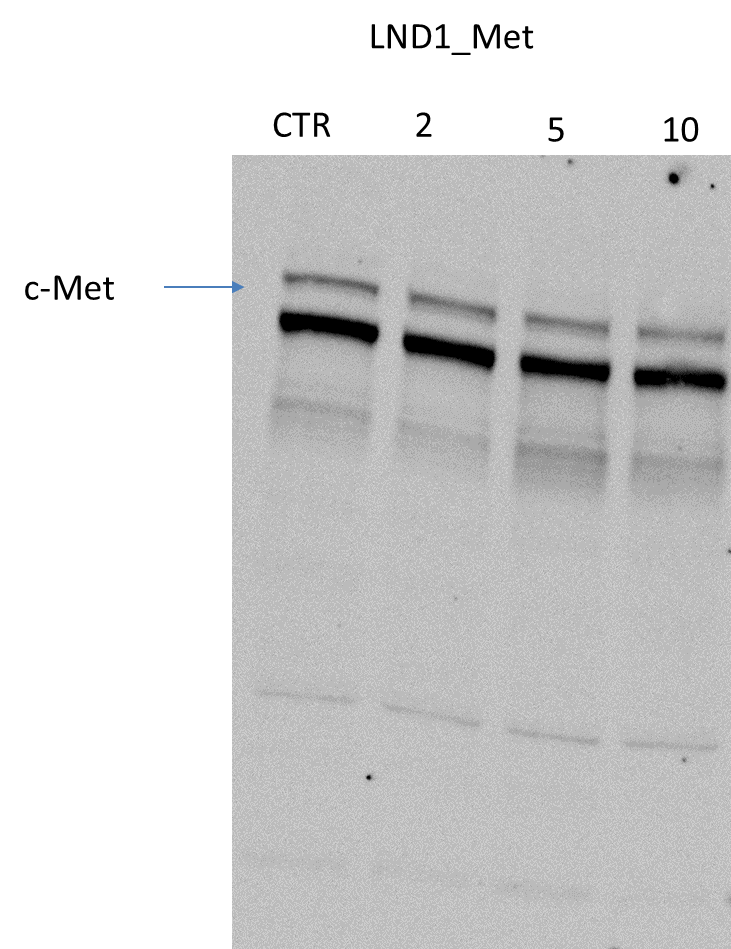

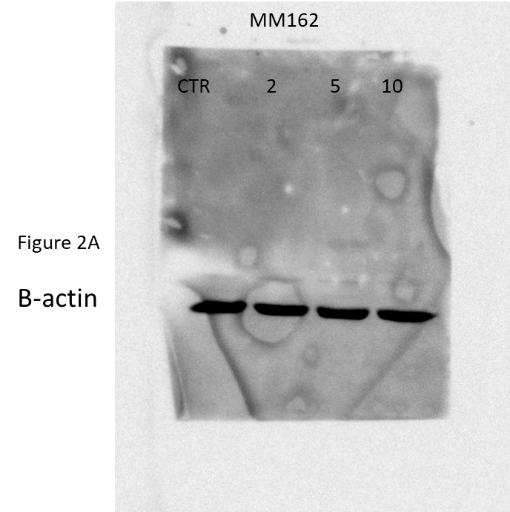


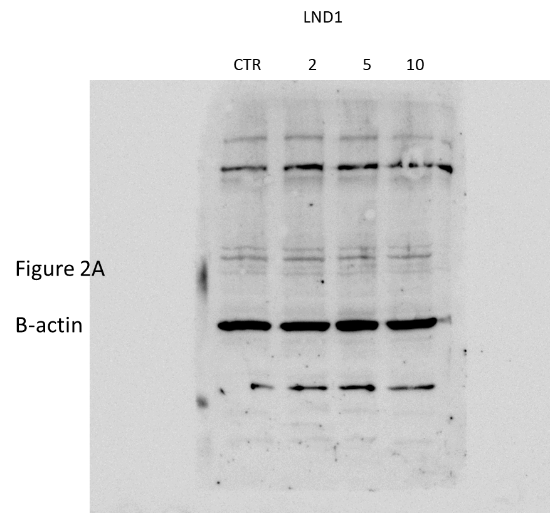


Figure S4: Uncropped images of the original western blots from western blot analysis shown in Figure 2A

**
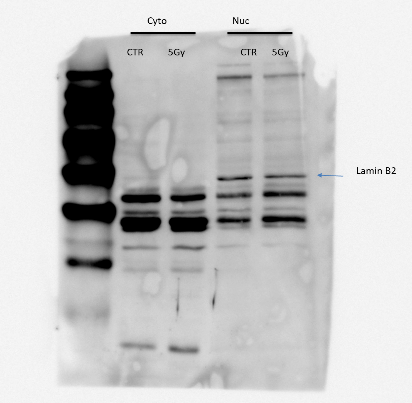

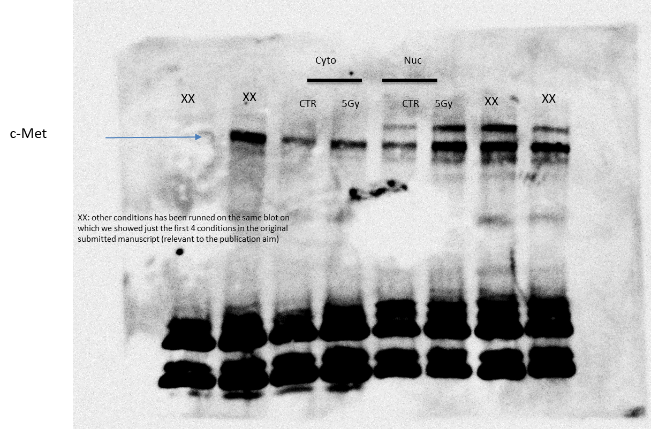
**

**
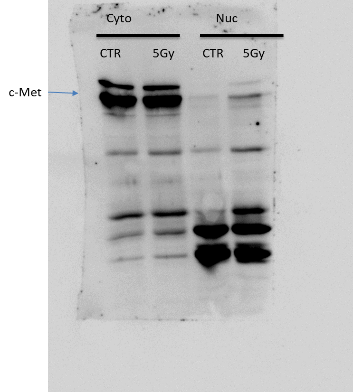

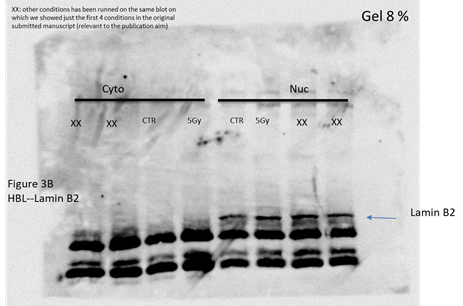
**

**
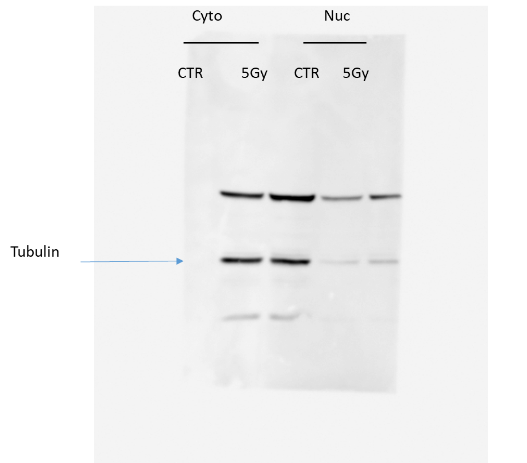
**

Figure S5: Uncropped images of the original western blots from western blot analysis shown in Figure 3B

**
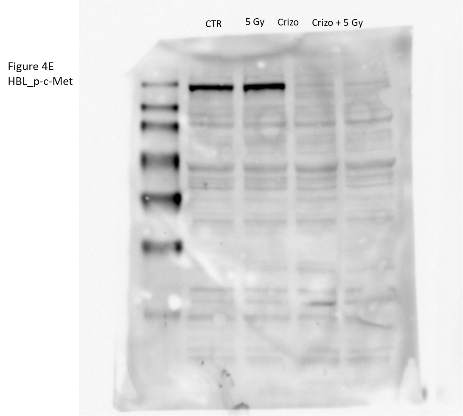

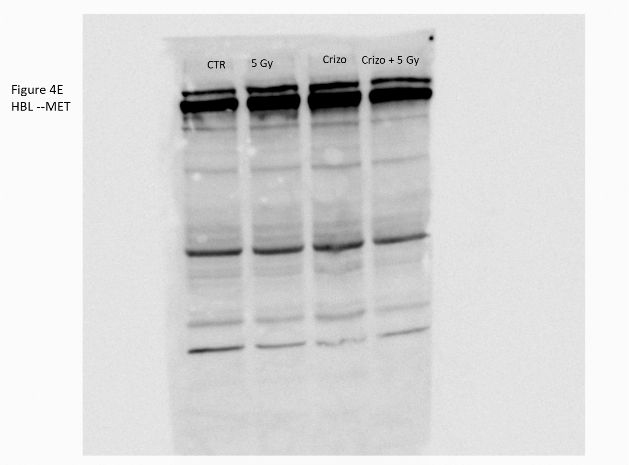
**


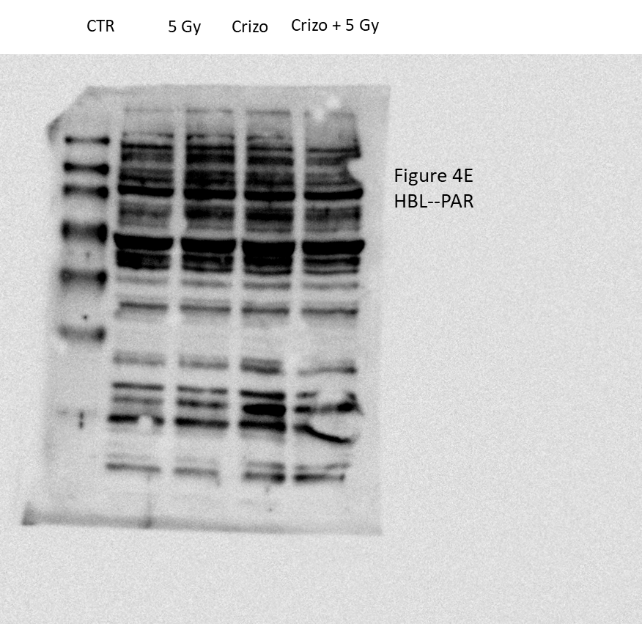

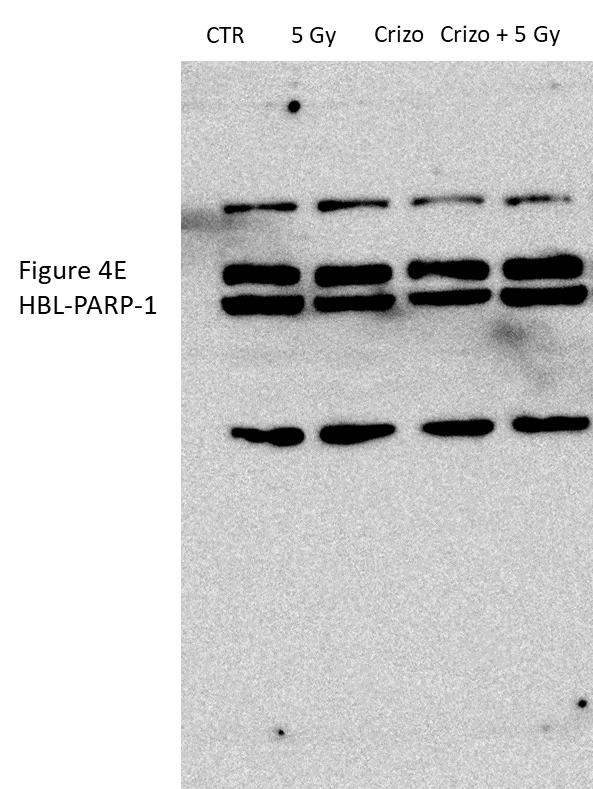


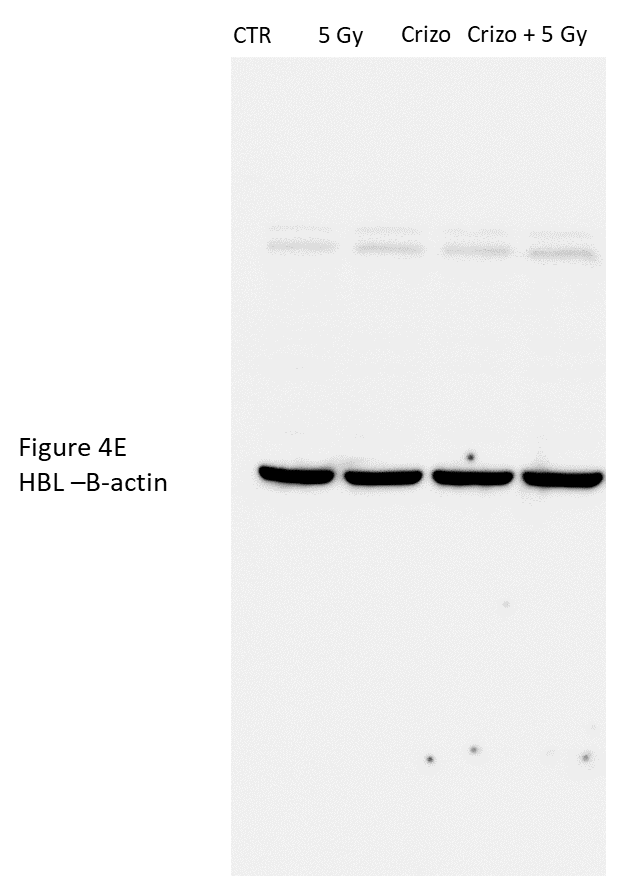


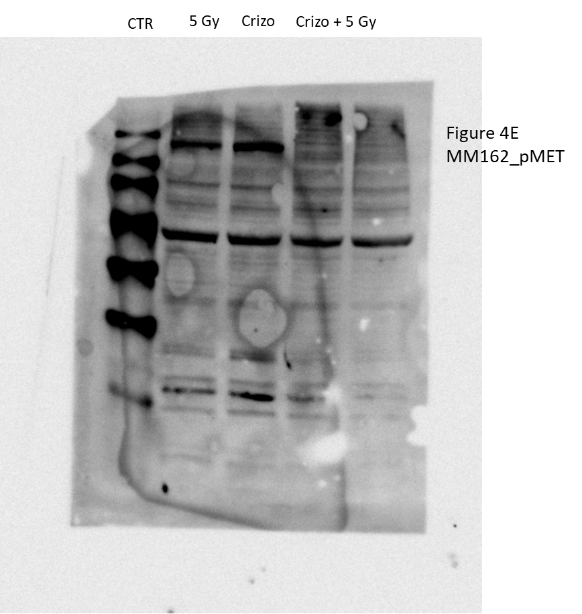

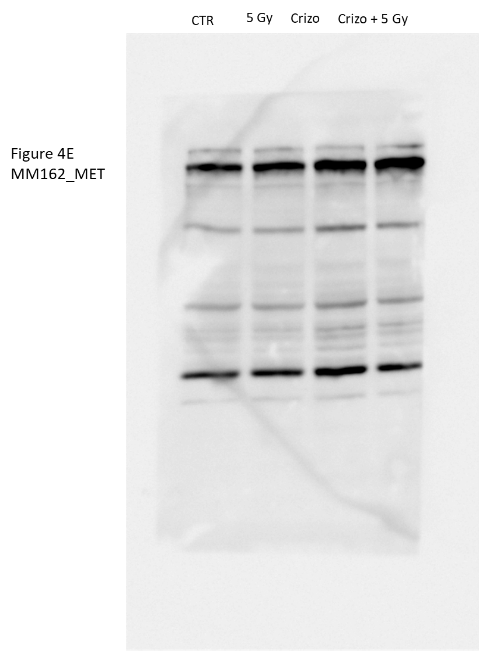


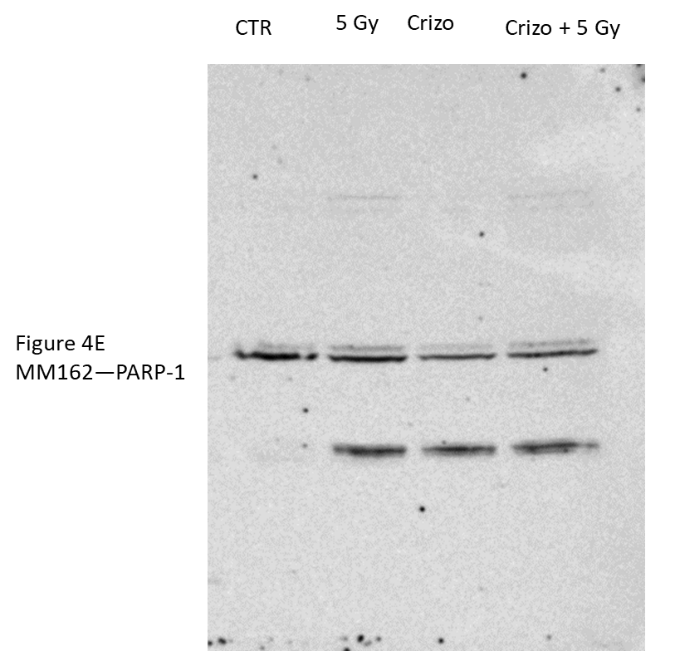

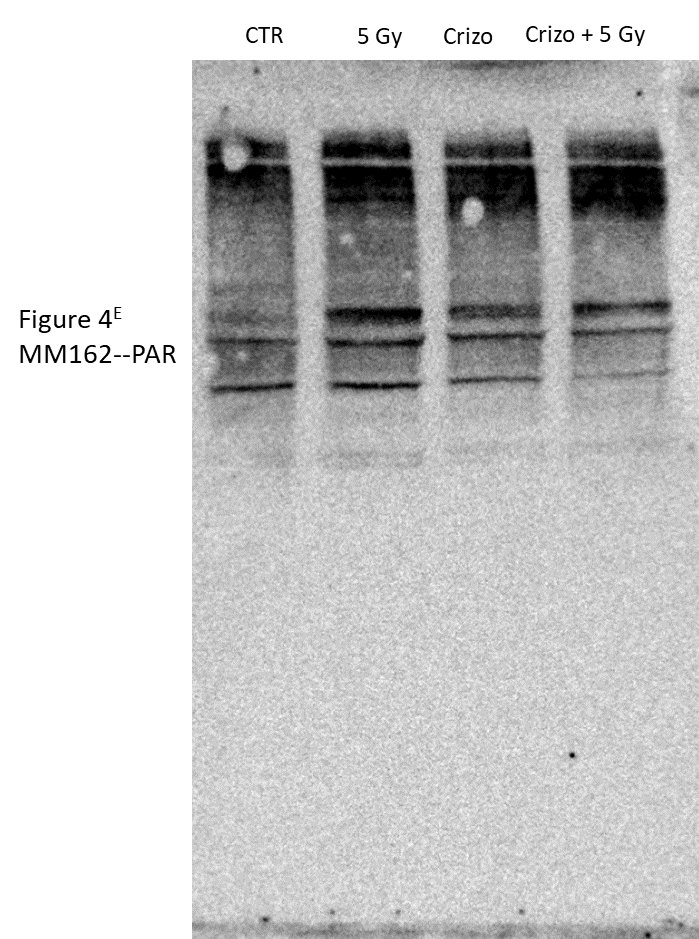


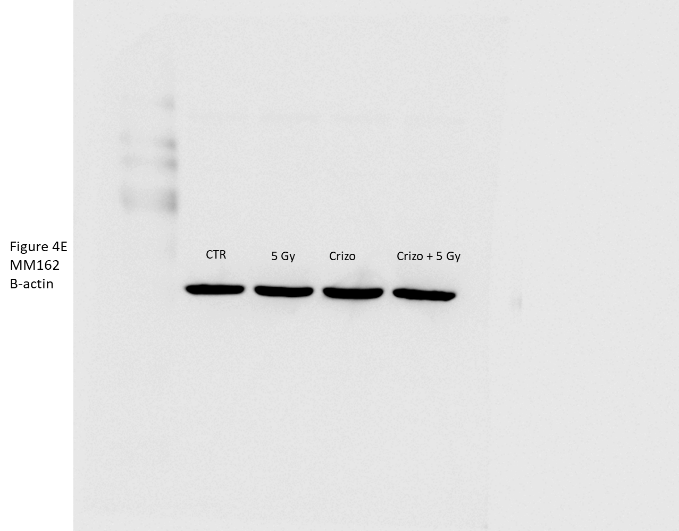


Figure S6: Uncropped images of the original western blots from western blot analysis shown in Figure 4E.
